# Research Progress on Anti-Inflammatory Mechanism of *Inula cappa*

**DOI:** 10.3390/ijms26051911

**Published:** 2025-02-23

**Authors:** Ningning Wu, Siqi Wang, Yuqian Zhang, Siming Wang

**Affiliations:** School of Basic Medical Sciences, Hebei University, Baoding 071000, China; 15512969626@163.com (N.W.); wangsiqi20220222@163.com (S.W.); zhangyuqian0903@163.com (Y.Z.)

**Keywords:** *Inula cappa*, anti-inflammatory, TLR2/MyD88/NF-KB signaling pathway

## Abstract

The incidence of various inflammatory diseases has remained high. *Inula cappa* is a kind of Chinese herbal medicine with a wide range of pharmacological uses and application value. It has anti-inflammatory, antibacterial, antioxidant, hepatoprotective and other pharmacological activities. The monomeric compounds that have been confirmed to have anti-inflammatory effects are luteolin, chrysoerilol, artemetin, chlorogenic acid, neochlorogenic acid, cryptchlorogenic acid, isochlorogenic acid A, isochlorogenic acid B, isochlorogenic acid C and 1,3-*O*-dicaffeoylquinic acid. This article introduces the relationship between *Inula cappa* and inflammation, the anti-inflammatory components of *I. cappa*, the modulation of each component on the inflammatory transduction signal pathway, and the TLR2/MyD88/NF-KB anti-inflammatory signaling pathway, providing a theoretical basis for anti-inflammatory research on and clinical medication using *Inula cappa*.

## 1. Introduction

Inflammation is a complex defensive response of the body to external damage. It stimulates cells to produce a variety of cytokines and regulates the balance between pro-inflammatory and anti-inflammatory systems. Inflammation plays an important role in protecting the body and defending against infections of foreign pathogens. It is generally believed that inflammation is a beneficial host defense system, but excessive and dysfunctional inflammatory responses can be harmful. In the dysregulated inflammatory response, complex inflammatory mediators, including pro-inflammatory cytokines and cytotoxic cytokines, are associated with the development of different diseases, including pneumonia [1], arthritis, chronic bronchitis, rheumatism [2] and even some neurodegenerative diseases, such as Alzheimer’s disease [3]. In recent years, the incidence of various inflammatory diseases has continued to high and is on the rise, even becoming a global disease [4]. The demand for safe and effective drugs to treat inflammatory diseases is rising.

**Figure 1:**
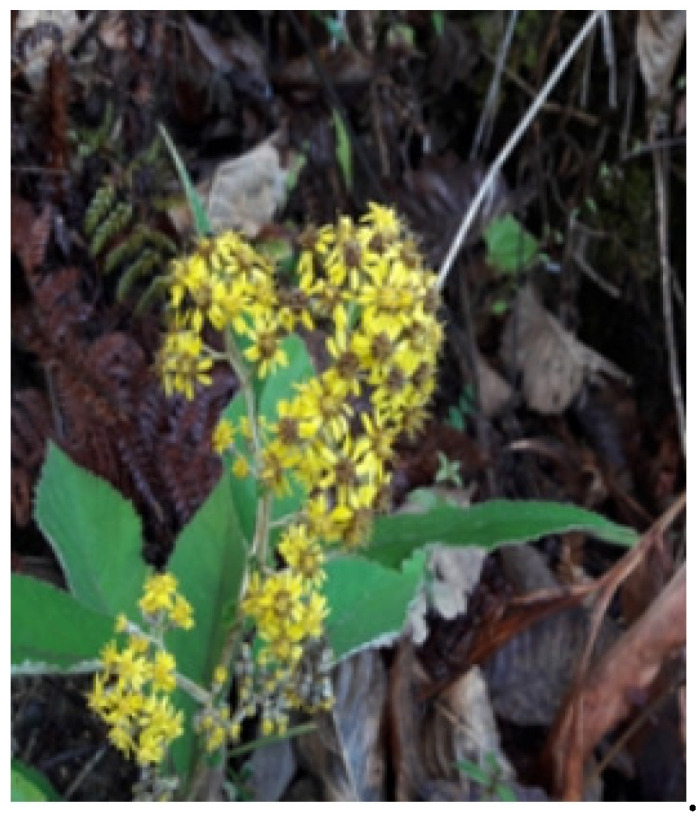
This is a picture of *Inula cappa*.

**Figure 2:**
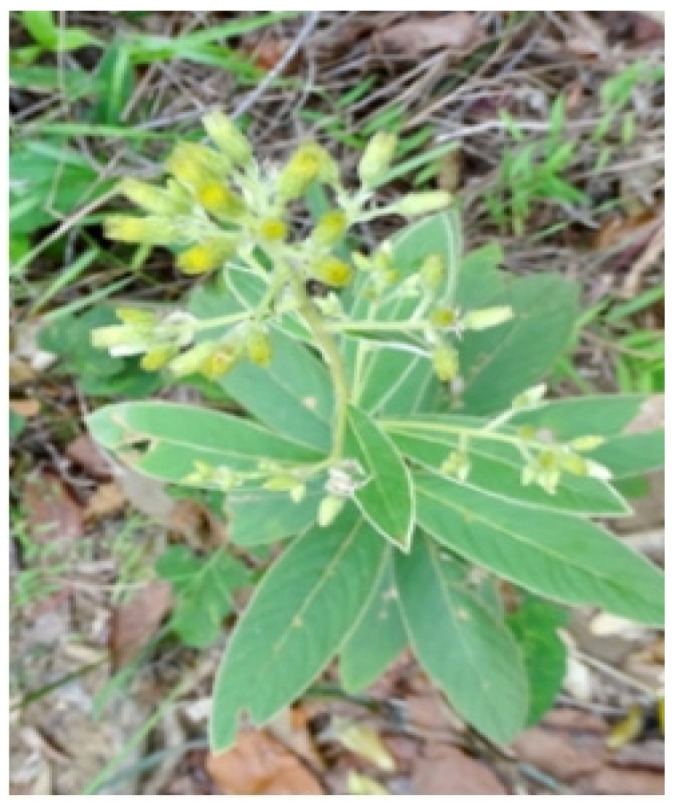
This is a picture of *Inula cappa*.

**Figure 3:**
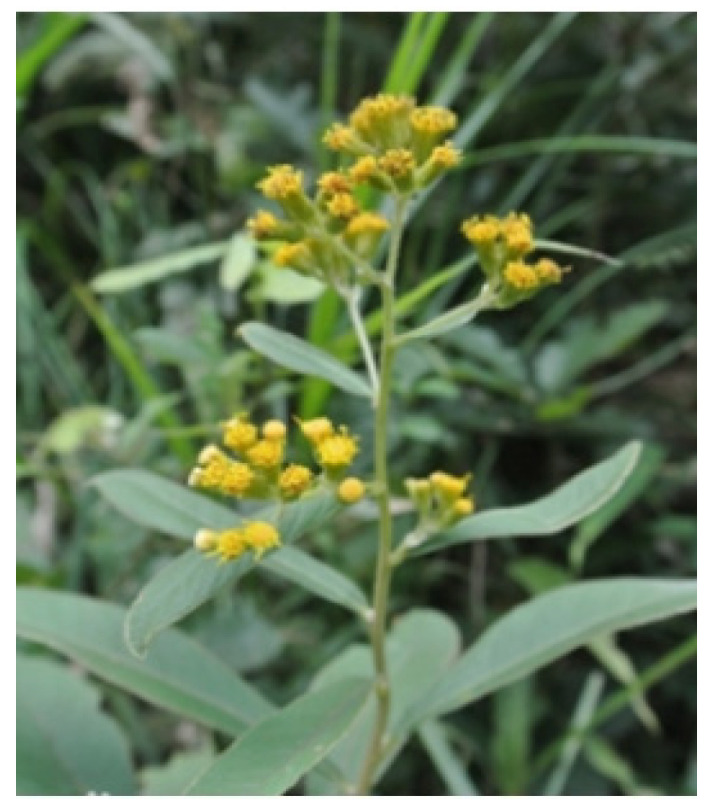
This is a picture of *Inula cappa*.

*Inula cappa* has a long history of medical use in China and has a wide range of pharmacological uses and application values. The plant is a subshrub, with stout rhizomes, erect multi-branched stems, 70–200 cm high, and white or light brown silky or cottony dense hairs. It has florets 4–5.5 mm long, marginal floret ligule short, with 3–4 lobes, or without ligule and with four staminodes; central floret tubular, with triangular-ovoid lobes distally; corolla hairs white, as long as tubular corolla, with more than 20 rough hairs. The flowering period is from June to October, and the fruit period is from August to December. The fruit is long and cylindrical, about 1.8 mm in length, with white, long sericeous hairs. Pictures of *Inula cappa* are shown in Figure 1, Figure 2 and Figure 3. All pictures are from the following website address: https://baike.baidu.com/item/%E7%BE%8A%E8%80%B3%E8%8F%8A/5009988#reference-2 (accessed on 19 January 2025).

More detailed information about *I. cappa* is shown in Table 1.

Modern studies have shown that *I. cappa* contains sesquiterpenes, inositols, triterpenes, flavonoids, caffeoylquinic acids and other chemical components, and the plant has a fundamental role in anti-inflammatory, antibacterial, antioxidant, liver protection and other pharmacological effects [7]. In this paper, the chemical constituents and mechanism of the anti-inflammatory effect of *Inula cappa* were studied and discussed in order to provide a theoretical basis for further research and the development and utilization of the herb.

## 2. Anti-Inflammatory Effect of *Inula cappa*

Modern pharmacological studies have shown that the extract of *I. cappa* has anti-inflammatory factors, which is bound to have a repair effect on inflammatory tissues and organs [11].

Mo Jiajia et al. measured the anti-inflammatory effect of the ethanol extract of the herb by the xylene-induced mouse ear swelling method, mouse abdominal capillary permeability hyperfunction method, acetic acid writhing method and hot plate method. The results showed that the ethanol extract of the plant root could significantly inhibit xylene-induced mouse ear swelling and acetic acid-induced capillary permeability hyperfunction in mice, and had a certain anti-inflammatory effect. The mechanism may be related to the improvement in local blood circulation, reduction in exudation and acceleration of absorption [5]. Based on the in vitro and in vivo inflammation model, Gong Zipeng found that the extract of the herb had anti-inflammatory activity [12]. Zhang Wanyou conducted an experimental study with the alcoholic extract of the herb roots and found that it has anti-inflammatory and analgesic effects, which provides a reference for the clinical use of the drug [13]. Kalola Jyoti et al. used the carrageenan-induced rat paw swelling method and cotton ball granuloma method to determine the anti-inflammatory and immunomodulatory activity of the root extract. The results showed that the methanol extract had the greatest inhibitory effect on rat paw swelling and had a significant inhibitory effect on rat cotton ball granuloma. The root extract of *Inula cappa* may inhibit chronic inflammation and acute inflammation by inhibiting the enzyme activity of arachidonic acid metabolic enzymes [14]. Huang Jing et al. found that the anti-inflammatory active ingredients in the extract were easily recognized and absorbed by inflammatory cells in vivo [15]. Wang Yunfei et al. found that the aqueous extract of the plant could effectively improve osteoarthritis [16]. Wang et al. found that the extract of the herb could effectively inhibit the release of NO from RAW264.7 cells stimulated by LPS in a certain range [17]. It was confirmed that the active ingredients of the herb could inhibit the secretion of TNF-α in addition to inhibiting the release of NO [18]. Various experimental studies have demonstrated that *Inula cappa* has certain anti-inflammatory effects.

## 3. Anti-Inflammatory Components of *Inula cappa*

So far, according to domestic and international research reports, the chemical constituents isolated and identified from the roots, stems, leaves and skins of *Inula cappa* mainly include sesquiterpenes, inositols, triterpenoids, flavonoids, caffeoylquinic acids, etc. [19,20,21].

*Inula cappa* has a strong inhibitory effect on a variety of inflammatory reactions. Sesquiterpenes, inositols, flavonoids and caffeoylquinic acids may be its anti-inflammatory active ingredients [7,22]. Sesquiterpenoids are the characteristic components of Inula plants. It has been found that most of the sesquiterpenoids isolated from Inula plants have obvious anti-inflammatory and anti-tumor effects [23,24]. Inositol is a cyclic polyol with six hydroxyl groups. After epimerization, nine isomers are formed. The most common ones are myoinositol (MI) and D-chiral inositol (DCI). Inositol has been shown to have the potential to treat polycystic ovary syndrome [25], inflammatory conditions [26], and hypoglycemia [27]. Flavonoids are a class of low-molecular natural plant components widely found in plants. Flavonoids are ubiquitous in Inula plants. Flavonoids have antibacterial, anti-inflammatory, antiviral and antioxidant effects [7]. Caffeoylquinic acid compounds are natural phenolic compounds that are synthesized by the condensation of quinic acid with one or more caffeic acids through an esterification reaction. They have antibacterial, anti-inflammatory, antiviral, and free radical scavenging effects, among others [28].

**Figure 4:**
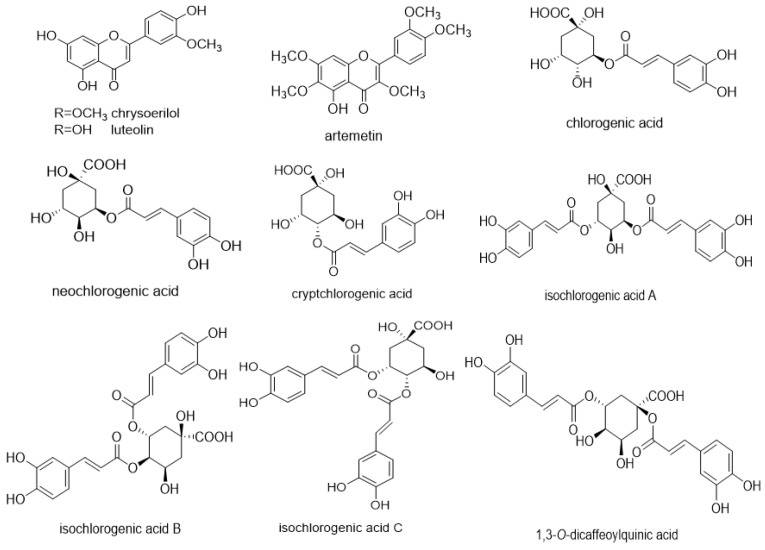
Monomer compounds with anti-inflammatory activity extracted from *Inula cappa* are luteolin, chrysoerilol, artemetin, chlorogenic acid, neochlorogenic acid, cryptchlorogenic acid, isochlorogenic acid A, isochlorogenic acid B, isochlorogenic acid C and 1,3-*O*-dicaffeoylquinic acid. The corresponding chemical structure formulas of each monomeric compound are also shown in the figure.

At present, the compounds with anti-inflammatory activity extracted from *I. cappa* have been reported to be luteolin, artemetin, chrysoerilol, chlorogenic acid, neochlorogenic acid, cryptchlorogenic acid, isochlorogenic acid A, isochlorogenic acid B, isochlorogenic acid C and 1,3-*O*-dicaffeoylquinic acid [15,18,29,30,31,32,33]. The structural formula of the related monomer compounds is shown in the following Figure 4. In addition, there are few studies on the anti-inflammatory mechanism of these 10 monomer compounds extracted from *I. cappa*. The modulation of these 10 monomeric compounds extracted from other plants on the inflammatory signal transduction pathway is shown in Table 2. It provides more ideas for the study of the anti-inflammatory mechanism of *I. cappa*.

## 4. Related Pathways of *Inula cappa’s* Anti-Inflammatory Effect

*Inula cappa* has a strong inhibitory effect on a variety of inflammatory responses. Caffeoylquinic acids, flavonoids, inositols and sesquiterpenes may be its anti-inflammatory active ingredients. These components have been shown to ameliorate inflammation by regulating signaling pathways, such as MAPK, TLR2 and NF-KB, and modulating the secretion of inflammatory factors such as TNF-α, IL-6, IL-1β and NO [61,62].

It has been proven by experiments that the extract of *Inula cappa* can reduce the inflammatory injury of pneumonia caused by Klebsiella pneumoniae by affecting the MAPK signaling pathway and the NF-KB signaling pathway [12]. In addition, another experimental result showed that the aqueous extract of *Inula cappa* may improve the inflammation of rats with severe pneumonia by inhibiting the TLR2/MyD88/NF-KB signaling pathway [63]. Therefore, the MAPK, NF-KB and TLR2 pathways are described in this article.

### 4.1. MAPK Pathway

Mitogen-activated protein kinase (MAPK) is a kind of serine/threonine protein kinase discovered by Sturgill and Ray in 3T3-L1 adipocyte extract in 1986, which is mainly distributed in the cytoplasm [64]. MAPK has a serine/threonine kinase domain, with different nitrogen terminal and carbon terminal regions on both sides. There are different additional domains, including the trans-activation domain (TAD), nuclear localization sequence (NLS) and conserved domain (C34) structural domain in ERK3/4 [65].

The MAPK pathway is a fundamental signal transduction pathway in mammalian cells. It is a key bridge connecting inside and outside the cell, which is stimulated by diverse extracellular signaling molecules (growth factors, neurotransmitters, cytokines, hormones, etc.). After the upstream activator protein binds to a specific receptor, it triggers a three-level enzymatic cascade reaction (MAPKKK-MAPKK-MAPK) [66]. MAPK kinase kinase (MAPKKK) is activated by mitogen-stimulated phosphorylation. On this basis, MAPKKK is phosphorylated to activate MAPK kinase (MAPKK). Finally, MAPKK phosphorylates MAPK and transmits signals to downstream response molecules through continuous activation. Ultimately, activated MAPK transduces extracellular stimuli into cells and their nuclei and causes cell responses, leading to proliferation, migration, differentiation, inflammation, stress, survival or apoptosis, thereby promoting the occurrence and development of diseases [67,68]. The related MAPK pathway kinases are shown in Table 3.

The MAPK superfamily can be divided into four members: extracellular signal-regulated kinase 1/2 (ERK1/2), c-Jun NH2-terminal kinase (JNK), p38 mitogen-activated protein kinase (p38MAPK) and ERK5. The most studied proteins in the MAPK pathway are ERK1/2, JNK and p38MAPK [69].

#### 4.1.1. ERK Signaling Pathway

ERK (extracellular signal-regulated kinase) pathway is a classical MAPK signal transduction pathway. ERK1/2 kinase is composed of ERK1 kinase and ERK2 kinase. ERK1/2 can be activated by various external signals such as cell growth factors, T hormones, G protein-coupled receptor ligands, osmotic pressure, neurotransmitters and neurotrophic factors [70].

The activation pathway of the ERK pathway is Ras→ Raf→ MEK1/2→ ERK1/2. After being activated by cytokines and other stimuli, Ras binds to Raf, and the activated Raf then moves from the cytoplasm to the cell membrane. MEK is a special bispecific kinase on the cell membrane. MEK binds to activated Raf and is activated by phosphorylation, resulting in ERK1/2 activation. Activated ERK1/2 translocates into the nucleus and activates downstream transcription factors and kinases [71]. The downstream related transcription factors are Ets-1, ATF-2, ElK-1, NF-KB, c-Fos and c-Myc. Cytoplasmic and nuclear kinases include MNKs, MPKAP-2, RSK and MSKs. This pathway plays a significant role in regulating gene transcription, cell growth, proliferation, cell cycle regulation, maintaining cell morphology, immune response, apoptosis and inflammatory response [43,67].

#### 4.1.2. JNK Pathway

In 1990, JNK (c-Jun N-terminal kinase) was first identified as a phosphorylated c-Jun kinase, hence the name c-Jun N-terminal kinase [72]. JNK was isolated from the liver of mice injected with cycloheximide, and there are three subtypes (JNK1, JNK2, JNK3). JNK1 and JNK2 are widely present in the body, and JNK3 is mainly expressed in brain tissue [66]. JNK is a kind of serine/threonine kinase that can be activated by various extracellular stimuli, including growth factors, cytokines and cellular stress, such as ultraviolet radiation, high osmotic pressure, heat shock and ischemia–reperfusion [73].

The activation of the JNK pathway is MEKK1-4, TAK, MLKs, ASKs → MKK4, MKK7 → JNK. In the JNK pathway, MEKK1-4, mixed-ligation kinases (MLKs), transforming growth factor-β-activated kinases (TAKs), apoptosis signal-regulated kinases (ASKs) in MAPKKK, MKK4 and MKK7 in MAPKK are activated. MKK4 and MKK7 are the bispecific kinases of JNK, but the way of activation is different. MKK4 is mainly activated by environmental stress, while MKK7 is mainly activated by cytokines. The activation mode of the JNK signaling pathway is that the activation of MEKK1-4 can phosphorylate MKK4 and MKK7. The activated MKK4 and MKK7 can activate JNK by phosphorylating the ThR and TyR sites of JNK [74]. JNK can activate different transcription factors (AP-1, c-Jun, ATF-2, ElK-1, c-Myc, p53, Bad, MLK2) and some members of the Bcl-2 family [75]. The JNK signaling pathway is involved in regulating a variety of cellular processes, including apoptosis, proliferation, transduction pathways, differentiation, inflammatory response and oxidative stress [69,76].

#### 4.1.3. P38MAPK Pathway

P38 mitogen-activated protein kinase (p38MAPK) was first discovered by Han et al. in 1994. It is one of the signaling pathways in the MAPK family that can be activated by various extracellular stimuli (cytokines, physiological stress, and ultraviolet osmotic pressure changes). It is a material pathway that mediates the survival and death of nerve cells [77,78]. There are four major subtypes of P38MAPK, including p38α, p38β, p38γ, and p38δ. P38α and p38β are widely distributed and can be expressed in almost all tissues and cells. The distribution of p38δ and p38γ is relatively tissue-specific.

The activation of P38MAPK depends on a typical three-level enzymatic cascade. The activation pathways of p38MAPK pathway is as follows: MLKs, ASK1, MEKKs, TAK→ MKK3, MKK4, MKK6→ p38MAPK. Ten MAP3Ks (ASK1, DLK, MEKK3, MEKK4, MLK3, TAK1, TAO1, TAO2, TPL2, ZAK1) contribute to the activation of P38 kinase, although some of them can also trigger other MAPK activation pathways, such as JNK [79]. MAP3K phosphorylates MKK3, MKK4 and MKK6 in MAP2K [79]. MKK3, MKK4 and MKK6 are known upstream-specific protein kinases of p38. Different p38 MAPK subtypes can be activated by different MKKs. MKK3 can activate p38α, p38γ and p38δ, MKK4 only activates p38α, and MKK6 is a common activator of different subtypes. Different subtypes can activate different downstream substrates, including other protein kinases, phospholipases and cytoskeleton-binding proteins. After being activated, p38MAPK enters the nucleus or transfers to other parts and regulates cell growth, cytoskeleton remodeling, apoptosis, inflammatory response and hyperalgesia by controlling the gene expression of various transcription factors [80].

### 4.2. NF-KB Pathway

Nuclear factor-KB (NF-KB) is a general name for a family of transcription factors. Its function is to coordinate a variety of physiological and pathological processes as a dimer. Members of the NF-KB family include p50, p52, RElA (p65), RElB and cREl. NF-KB can be divided into two groups. One group is p50 and p52, which are produced, respectively, by the cleavage of p105 and p100 precursors. The other group was RElA (p65), RElB and cREl, without precursors. The NF-KB protein refers to the NF-KB1 dimer protein formed by the p65/p50 subunit and the NF-KB2 dimer protein formed by the RElB/p52 subunit.

In the resting state, it binds to the inhibitor of NF-KB (IKB) and exists in the cytoplasm in an inactive form [81]. IKB is a complex composed of IKKα and IKKβ and the basic modulator (NEMO) of NF-KB [82].

The classical NF-KB signaling pathway is induced by a variety of immune mediators. When activated by upstream stimulation signals such as LPS, TNF-α, IL-1 and IL-6, IKB kinase (IKK) is activated and can activate IKBα/β phosphorylation at specific N-terminal serine residues, causing IKB to be phosphorylated and degraded, which in turn enables p65/p50 dimers normally present in the cytoplasm to be transferred to the nucleus and bind to the KB sequence in the promoter region of the relevant target gene sequence to exert its biological effects [82,83].

The bypass pathway mainly refers to the activation of NF-KB containing pl00 or pl05 dimers. In some cells, after being stimulated by extracellular signals, IKKa is phosphorylated and activated under the action of NF-KB-induced kinase (NIK) and further activates p100, resulting in the phosphorylation of p100 and subsequent cleavage by corresponding enzymes to form an active RElB/p52 complex. The RElB/p52 complex enters the nucleus of the cell and binds to the target gene to regulate the expression of the corresponding gene [84,85].

In addition, the mechanism of UV-activated NF-KB is different from the above pathways. Ultraviolet light causes the phosphorylation of the C-terminus of IKBα by activating casein kinase 2 (CK2), which leads to the ubiquitination and degradation of IKBα. The activation of CK2 is not dependent on IKK but through P38 mitogen-activated protein kinase, so the p38-CK2-IKBα pathway is also one of the ways to activate NF-KB [86].

NF-KB protein is a member of the transcription factor family, which is stimulated by chemokines, ECM degradation products, stress-related factors and pro-inflammatory cytokines. More and more studies have shown that NF-KB is a transcription factor involved in the regulation of immune and inflammatory responses. The activation of NF-KB is accompanied by inflammatory responses, such as asthma, rheumatoid arthritis, psoriasis, and enteritis. In addition, NF-KB also plays an important regulatory role in expanding inflammatory responses [87]. Therefore, inhibiting the activation of the NF-KB signaling pathway is of great significance for the prevention and treatment of inflammatory or immune diseases [38].

### 4.3. Toll-TLR2 Pathway

#### 4.3.1. Introduction of Toll-like Receptors

Toll-like receptors (TLRs) are usually a group of evolutionarily conserved type I transmembrane proteins, which is a family of pattern recognition receptors, mainly involved in cellular immune response and inflammatory response. At present, 13 different TLRs have been found in mammals, of which 11 TLRs play a role in the human body [88]. TLRs exist in more than 20 kinds of cells, among which TLR2 and TLR4 are the most studied members [89].

TLRs are composed of three parts: extracellular, cytoplasmic and transmembrane regions, which consist of an extracellular domain at the N-terminal end of the membrane, a single transmembrane structural domain and an intramembrane C-terminal structural domain [90]. The N-terminal extracellular domain contains a leucine-rich repeat (LRR) and selectively recognizes PAMP, and each TLR can recognize a specific molecular pattern. The transmembrane region has a simple structure and is composed of a cysteine-rich domain that connects the intracellular and extracellular regions of TLRs across the cell membrane. The C-terminal domain, also known as the cytoplasmic domain, contains an evolutionarily conserved Toll/IL-1 receptor (TIR) homology domain, and each TLR can recognize a specific molecular pattern and is responsible for signal transduction [91].

The TLR signaling pathway is divided into MyD88-dependent and MyD88-independent signaling pathways. MyD88 is a cytoplasmic soluble protein that consists of three functional regions: the N-terminal death domain, the intermediate region and the C-terminal homeodomain [92]. The MyD88-dependent signaling pathway mainly mediates the production of cytokines. In addition to TLR3, all of the currently identified TLR family members can activate the MyD88-dependent pathway by binding to agonists, thereby activating downstream signaling molecules, causing cascades and activating related signaling pathways [89].

TLRs recruit adaptor protein myeloid differentiation factor 88 (MyD88) to form a complex with IL-1 receptor-associated kinase (IRAK) family molecules. After the formation of the complex, IRAK4 phosphorylates IRAK1. Phosphorylated IRAK1 binds to tumor necrosis factor receptor-associated factor-6 (TRAF6), and then ubiquitination initiates nuclear factor kappa-B (NF-KB), cAMP response element-binding protein (CREB) and activating protein-1 (AP-1), thereby triggering an inflammatory response. The MyD88-independent pathway is also known as the TRIF pathway. TLRs recruit and activate TIR domain-containing adaptor-inducing interferon β (TRIF) and utilize TRAF3 and TRAF6 to activate interferon-regulatory factor (IRF) 3 and 7 and NF-KB. This leads to the expression of type I interferons and the production of inflammatory cytokines [93,94].

The specific process of the signaling pathway is as follows [95].

MyD88 signaling pathway: TLRs recruit the adaptor protein MyD88 to its TIR domain. MyD88 contains two domains: one binds to the TIR domain, and the other is called the death domain to recruit IRAK 1,2,4 proteins. IRAK protein recruits TRAF6 to the receptor complex, and IRAK4 phosphorylates IRAK1 after the formation of the complex. Then, phosphorylated IRAK1 and TRAF6 are dissociated from the complex and form a complex with TAK1 (TGF-β-activated kinase 1), TAB1 and TAB2 on the plasma membrane, thereby phosphorylating all three. Subsequently, IRAK1 is degraded on the plasma membrane, and the remaining complex (composed of TRAF6, TAK1, TAB1 and TAB2) is translocated to the cytosol, resulting in TRAF6 ubiquitination and TAK1 activation. Activated TAK1 acts on the IKB kinase (IKK) complex (composed of IKKα, IKKβ and IKKγ) to activate IKK, thereby phosphorylating IKB and leading to IKB ubiquitination and proteasome degradation, and then releases NF-KB to the nucleus, inducing the transcription of a variety of related proteins, including various pro-inflammatory cytokines, such as IL-6, IL-12p40 and TNF family-related molecules. AK1 can also activate the MKK3/6-p38 signaling cascade and the MKK4/7-Jun N-terminal kinase (JNK) pathway, leading to the production of CREB and AP-1, respectively.

TRIF signaling pathway: TLRs recruit TRIF to the TIR domain, and TLR4 can also recruit TRAM (TRIF-related adaptor molecule) to TIR. Subsequently, IKKε, TBK1 and TRAF3 are recruited into TRIF/TIR or TRAM/TRIF/TIR complexes, and TBK1 phosphorylated IRF3 and IRF7, which combined with p300 and CBP (CREB binding protein) to activate interferon-induced gene expression. TRIF can also bind to TRAF6 and produce pro-inflammatory cytokines by activating NF-KB.

#### 4.3.2. TLR2 Pathway

TLR2 is one of the important members of the Toll-like receptor family, which is involved in the immune response and inflammatory response of cells, accompanied by the occurrence and development of various diseases. TLR2 usually forms heterodimers with TLR1 or TLR6. The TLR2-TLR1 heterodimer can specifically recognize triacylglycerols of Gram-negative bacteria and mycoplasma, while the TLR2-TLR6 heterodimer can specifically recognize diacylglycerols of Gram-positive bacteria and mycoplasma. In addition, TLR2 can work together with other co-receptors on the cell surface. These co-receptors include CD36, which mediates the sensing of some agonists of TLR2 together with TLR2-TLR6 heterodimers [96].

The TLR2 pathway includes the classical TLR2-MyD88-NF-KB signaling pathway, the non-classical TLR2-PI3K/AKt-NF-KB and other pathways.

TLR2 signaling is mainly mediated by the MyD88-dependent pathway. After TLR2 binds to the adaptor protein molecule MyD88, it activates the downstream target gene protein molecules IRAK-4, IRAK-1, TRAF6, IKK-β, TGF-β, TAK1, IKK-γ, NF-KB, etc., forming the TLR2-MyD88-NF-KB pathway. After the interaction of TLR2 and its related adaptor proteins, the IRAK complex is activated to recruit TRAF6. Activated TRAF6 triggers the activity of the transforming growth factor β-activated kinase 1 (TAK1)/TAK1-binding protein (TAB) complex and stimulates the activation of MAPK and nuclear factor KB inhibitor kinase complexes (IKK1,2 and IKK-γ), also known as NF-KB Essential Modulator (NEMO). The MAPK family involved includes JNK and p38MAPK. The IKK complex promotes the nuclear translocation of NF-KB. This leads to the production of pro-inflammatory cytokines by activator protein-1 (AP-1) and NF-KB, thereby controlling inflammation and regulating cell survival and proliferation [97].

The specific process of non-classical TLR2-PI3K/AKt-NF-KB is as follows. After being activated by endogenous DAMPs released by other cells, TLR2 can transmit signals to phosphatidylinositol-3-kinase (PI3K) and catalyze the phosphorylation of phosphatidylinositol diphosphate (PIP2) to phosphatidylinositol triphosphate (PIP3). PIP3 binds to protein kinase D1 (PKD1) and protein kinase B (PKB/AKt) containing the PH domain to promote PKD1 to phosphorylate 308 serine of AKt. Like TAK-1, activated AKt can also phosphorylate a variety of proteins, such as the IKK complex, thereby activating the NF-KB signaling pathway and the transcription of a variety of target genes [98].

## 5. Conclusions and Discussion

Various experiments have shown that *Inula cappa* has a strong inhibitory effect on a variety of inflammatory reactions. Caffeoylquinic acids, flavonoids, inositols and sesquiterpenes in the plant may contain anti-inflammatory active ingredients. The specific anti-inflammatory components are known to be luteolin, artemetin, chrysoerilol, chlorogenic acid, neochlorogenic acid, cryptchlorogenic acid, isochlorogenic acid A, isochlorogenic acid B, isochlorogenic acid C and 1,3-*O*-dicaffeoylquinic acid. *Inula cappa* can alleviate inflammation by harmonizing MAPK, TLR2 and NF-KB signaling pathways and regulating the secretion of inflammatory factors such as TNF-α, IL-6, IL-1β and NO.

At present, there are few studies revealing the anti-inflammatory active components of *Inula cappa* and how this herb affects the potential targets of inflammation. The anti-inflammatory effects and mechanisms of other components of this herb are not deep enough.

More than 100 monomeric compounds have been extracted from *I. cappa* [11]. The anti-inflammatory substances in *I. cappa* are not only the 10 species listed in this paper; more in-depth research needs to be conducted on the other extracted components to prove whether they have anti-inflammatory effects. In addition, there are more studies on the modulation targets of the 10 monomer compounds extracted from other herbal medicine plants, providing a certain idea for research on the anti-inflammatory targets of *I. cappa*. However, to determine whether the anti-inflammatory targets of these 10 monomer compounds extracted from *I. cappa* and extracted from other plants are consistent, further experimental studies are needed.

Therefore, in the future, it is significant to further explore the anti-inflammatory active components of *Inula cappa*, the mechanisms and the specific targets of action to provide ideas for subsequent research and lay a foundation for its development and utilization.

## Figures and Tables

**Table 1:** Detailed information about synonyms, growing environment, distribution areas, medicinal parts, medicinal value and folk medication experience of *Inula cappa*.

*Inula cappa*	Detailed Information
Synonyms	Zhuerfeng (Guangxi), Shanbaizhi, Yangerfeng (Guizhou), Baimianmaozigu (Guangdong), Bainiudan, Wamaoxiang (Sichuan), Baimianfeng (Jiangxi), Zhuangniulang, Bamianfeng (Zhejiang), Nahan (Xishuangbanna), Yalangnong (Dehong)
Growing environment	It grows mainly in subtropical and tropical low mountains and subalpine regions with humid or dry conditions. It is also commonly found in wastelands, shrubs or grasslands as well as acidic soil, including sand and clay [5].
Distribution areas	It is found in Sichuan, Yunnan, Guizhou, Guangxi, Guangdong, Jiangxi, Hunan, Fujian and Zhejiang in China, and is also distributed in Vietnam, Myanmar, Thailand, Malaysia and India [6].
Medicinal parts	The whole grass or root of this plant can be used for medicine. The medicinal parts of ‘Xishuangbanna Dai Medicine Records’, ‘Chinese Dai Medicine Color Atlas’ and ‘Chinese Materia Medica (Dai Medicine Volume)’ are all roots and whole herbs. In the 2005 edition of ‘Yunnan Provincial Standards of Chinese Medicinal Materials (Dai Medicine)’, only roots are used as medicines. ‘Guizhou Miao medicine research and development’ records that the whole grass of *Inula cappa* is a common medicinal material of the Miao nationality.
Medicinal value	The herb is spicy, slightly bitter, warm; enters into the liver, lung, spleen, stomach; and has the effect of relieving cold, purging the liver, clearing heat and relieving dysentery [7]. It is used for cool wind colds, palpitations, hot flashes, irregular menstruation, epigastric pain, rheumatic arthralgia, neuropathic headache, asthma, furuncle abscess and other symptoms.
Folk medication experience	The herb can be used internally and externally, which is applied to Juhuang Shangqing Tablets [8], Pearl Dropping Pills [9], Shuanghou Bitong Granules [10] and other preparations. According to the record of ‘Chinese ethnomedicine’, in addition to the Han nationality, the Dai, Dong, Jingpo, Lahu, Lisu, Miao, Yi, Wa, Zhuang and other ethnic groups in southern China have rich experience in medicinal use. The plant is commonly used as anti-inflammatory and detumescence medicine in Wenshan, Xichou, and other counties of Yunnan. In Guizhou Miao nationality, *Inula cappa* is mainly used to disperse wind and clear heat, with detoxifying and detumescent effects, and is used for cold and fever, sore throat and swelling, rheumatic swelling and pain.

**Table 2:** Modulation of the inflammation transduction signal pathways by luteolin, chrysoerilol, artemetin, chlorogenic acid, neochlorogenic acid, cryptchlorogenic acid, isochlorogenic acid A, isochlorogenic acid B, isochlorogenic acid C and 1,3-*O*-dicaffeoylquinic acid.

Monomeric Compound	Modulation Target	References
Luteolin	Positive: SOCS3, Nrf2, SOD, CAT, GPx, GSH, HO-1 Negative: TNF-α, IL-6, COX-2, iNOS, MMPs, IL-1β, IL-18	[34,35,36]
Chrysoerilol	Negative: Ser536, Tyr705, iNOS, COX-2, IL-6, IL-1β, TNF-α, PGE2	[37,38]
Artemetin	Negative: TNF-α, IL-1β	[39]
Chlorogenic acid	Negative: TNF-α, IL-1β, IL-6, IFN-γ, MCP-1, MIP-1α, COX-2, PGE2.19, KAT2A, IL-8,	[40,41,42,43]
Neochlorogenic acid	Positive: Nrf2, HO-1, NQO-1 Negative: iNOS, COX-2, TNF-α, IL-1β, IL-6, IL-1β, ROS, PGE2	[44,45,46]
Cryptchlorogenic acid	Positive: IL-10, Nrf2, HO-1, Negative: iNOS, TNF-α, IL-1β, IL-6, IL-8, COX-2	[47,48]
Isochlorogenic acid A	Positive: IκBα, Negative: MMP13, IL-1β, IL-23, IL-17, p-JNK	[49,50]
Isochlorogenic acid B	Positive: BDNF, GPR161, TMEM59 Negative: IL-6, TNF-α, p-P65, TNF-α, IL-1β, COX-2, iNOS	[51,52]
Isochlorogenic acid C	Negative: iNOS, COX-2, IL-1β, ROS, TNF-α, PGE2, MMP, ADAMTS-4	[53,54,55,56]
1,3-*O*-dicaffeoylquinic acid	Positive: Nrf2, IκB-α, HO-1 Negative: MCP-1, TNF-α, IL-6, NLRP3, IL-1β, IL-18, ROS, iNOS	[57,58,59,60]

**Table 3:** Specific information about MAPK pathway-related kinases, including the full name of the kinase, the subtribe of the kinase and the number of subfamilies.

Kinase	Full Name	Subtribe	Number of Subfamilies
MAPK	Mitogen-activated protein kinase	ERK subtribe: ERK1, ERK2, ERK3, ERK4 P38 subtribe: P38α, P38β, P38γ, P38δ JNK subtribe: JNK1, JNK2, JNK3, ERK5	12
MAPK kinase	MAP kinase kinase, MKK, MAPKK	MEK1, MEK2, MEK5 MKK3, MKK4, MKK6, MKK7	7
MAPK kinase kinase	MAP kinase kinase kinase, MKKK, MAPKKK	Raf subtribe: A-Raf, B-Raf, Raf1 (c-Raf) MEKK subtribe: MEKK1, MEKK2, MEKK3, MEKK4 The third subtribe: ASK1, Tpl2 The fourth subtribe: MST (mammalian sterile 20-like) MUK (MAPK upstream kinase) MOS (molony sarcoma oncoprotein) SPRK, TAK1	14

## Data Availability

No new data were created or analyzed in this study. Data sharing is not applicable to this article.
